# Comparison of perioperative analgesia using the infiltration of the surgical site with ropivacaine alone and in combination with meloxicam in cats undergoing ovariohysterectomy

**DOI:** 10.1186/s12917-020-02303-9

**Published:** 2020-03-16

**Authors:** Gabriel de O.L. Carapeba, Isabela P. G. A. Nicácio, Ana Beatriz F. Stelle, Tatiane S. Bruno, Gabriel M. Nicácio, José S. Costa Júnior, Rogerio Giuffrida, Francisco J. Teixeira Neto, Renata N. Cassu

**Affiliations:** 1 Postgraduate Program in Animal Science, Universidade do Oeste Paulista (UNOESTE), Presidente Prudente, Brazil; 2 Department of Veterinary Surgery and Anesthesiology, Faculdade de Medicina Veterinária, Universidade do Oeste Paulista (UNOESTE), Presidente Prudente, Brazil; 3 Department of Veterinary Surgery and Animal Reproduction, Faculdade de Medicina Veterinária e Zootecnia, Universidade Estadual Paulista (UNESP), Botucatu, Brazil

**Keywords:** Analgesia, Feline, Infiltration, Local anesthetic, Non-steroidal anti-inflammatory drug

## Abstract

**Background:**

Infiltration of the surgical site with local anesthetics combined with nonsteroidal anti-inflammatory drugs may play an important role in improving perioperative pain control. This prospective, randomized, blinded, controlled clinical trial aimed to evaluate intraoperative isoflurane requirements, postoperative analgesia, and adverse events of infiltration of the surgical site with ropivacaine alone and combined with meloxicam in cats undergoing ovariohysterectomy. Forty-five cats premedicated with acepromazine/meperidine and anesthetized with propofol/isoflurane were randomly distributed into three treatments (*n* = 15 per group): physiological saline (group S), ropivacaine alone (1 mg/kg, group R) or combined with meloxicam (0.2 mg/kg, group RM) infiltrated at the surgical site (incision line, ovarian pedicles and uterus). End-tidal isoflurane concentration (FE’ISO), recorded at specific time points during surgery, was adjusted to inhibit autonomic responses to surgical stimulation. Pain was assessed using an Interactive Visual Analog Scale (IVAS), UNESP-Botucatu Multidimensional Composite Pain Scale (MCPS), and mechanical nociceptive thresholds (MNT) up to 24 h post-extubation. Rescue analgesia was provided with intramuscular morphine (0.1 mg/kg) when MCPS was ≥6.

**Results:**

Area under the curve (AUC) of FE’ISO was significantly lower (*P* < 0.0001) in the RM (17.8 ± 3.1) compared to S (23.1 ± 2.2) and R groups (22.8 ± 1.1). Hypertension (systolic arterial pressure > 160 mmHg) coinciding with surgical manipulation was observed only in cats treated with S and R (4/15 cats, *P* = 0.08). The number of cats receiving rescue analgesia (4 cats in the S group and 1 cat in the R and RM groups) did not differ among groups (*P* = 0.17). The AUC of IVAS, MCPS and MNT did not differ among groups (*P* = 0.56, 0.64, and 0.18, respectively). Significantly lower IVAS pain scores were recorded at 1 h in the RM compared to the R and S groups (*P* = 0.021–0.018). There were no significant adverse effects during the study period.

**Conclusions:**

Local infiltration with RM decreased intraoperative isoflurane requirements and resulted in some evidence of improved analgesia during the early postoperative period. Neither R nor RM infiltration appeared to result in long term analgesia in cats undergoing ovariohysterectomy.

## Background

There is increasing evidence that the use of multiple analgesics that act by different mechanisms (multimodal analgesia), with the objective to obtain additive or synergistic effects, is an effective method for surgical pain management [1]. The beneficial effects of intraperitoneal/incisional administration of local anesthetics, as part of a multimodal analgesia protocol, are well known in small animal practice [2–4].

Infiltration of the surgical site with local anesthetics is a simple, safe, and low-cost technique, which may improve perioperative analgesia due to inhibition of noxious impulse transmission at the wound site, resulting in anesthetic and analgesic-sparing effects [5–8]. In cats, reports of the effectiveness of infiltration of the surgical wound with local anesthetics are limited [5, 7, 8]. Recently, one study reported that intraoperative infiltration of bupivacaine in specific anatomical areas was an effective method for early postoperative pain control in cats undergoing ovariohysterectomy [8]. Until now, no studies have reported the effects of ropivacaine as part of a multimodal analgesia protocol in cats. Ropivacaine has a relatively fast onset (10–15 min) and a prolonged duration of action (90–360 min), with less risk of cardiac and systemic toxicity than bupivacaine [9]. The lower toxicity of ropivacaine represents an advantage, especially for cats, because this species appears more susceptible than dogs to the toxic effects of local anesthetics [10]. In isoflurane-anesthetized dogs undergoing ovariohysterectomy, the analgesic effects of intraperitoneal instillation of ropivacaine were considered similar to those provided by an equivalent dose of bupivacaine [11]. Intratesticular and incisional ropivacaine decreased intraoperative isoflurane requirements in dogs undergoing orchiectomy [12].

Recently, it has been suggested that infiltration of the surgical site with local anesthetics and non-steroidal anti-inflammatory drugs (NSAIDs) could play an important role in perioperative analgesia [13, 14]. Combination of local anesthetics and NSAIDs can produce effective analgesia by a dual mechanism, including a direct inhibition of noxious impulses from the site of injury and a reduced local expression of mediators able to sensitize nociceptors of afferent fibers, particularly prostaglandins [13]. Using a rat laparotomy model, previous studies reported pronounced analgesic benefits after the infiltration of the surgical wound with the combination of different NSAIDs with levobupivacaine and epinephrine [14, 15].

Among the NSAIDs used in cats, meloxicam is a preferential cyclooxygenase-2 inhibitor that has been widely used by subcutaneous and oral routes in the control of postoperative pain following ovariohysterectomy [4, 16, 17]. Nevertheless, there are no published studies focused on the administration of meloxicam or other NSAIDs into the surgical site combined with local anesthetics for perioperative pain relief in dogs or cats.

The aim of this study was to investigate the effects of infiltration of the surgical site with ropivacaine alone or combined with meloxicam on isoflurane requirements for maintaining anesthesia, postoperative pain, and morphine consumption. A secondary objective was to investigate the adverse effects of both analgesic protocols. The hypothesis was that the addition of meloxicam to ropivacaine would improve perioperative pain control by decreasing isoflurane requirements for maintaining anesthesia and by inducing prolonged postoperative analgesia following ovariohysterectomy in cats.

## Results

From the 50 cats initially enrolled in the study, five were excluded due to aggressiveness (*n* = 3) and pregnancy (*n* = 2). There were no significant differences between groups in age, body weight, body condition score, dose of propofol, duration of anesthesia and surgery, and time to extubation and recovery (*P* > 0.05) (Table 1).

**Table 1:** Demographic data, dose of propofol, area under curve (AUC) values for end-tidal isoflurane concentration (FE’ISO), and procedural times (mean ± standard deviation) of cats undergoing ovariohysterectomy treated with local infiltration with saline solution (S, *n* = 15), ropivacaine (R, *n* = 15) and ropivacaine/meloxicam (RM, *n* = 15)

Variables	Group			
	S	R	RM	*P* value
Body weight (kg)	2.7 ± 0.3	2.6 ± 0.5	2.4 ± 0.4	0.63
Age (months)	15.8 ± 7.5	17.4 ± 7.7	15 ± 8	0.68
Propofol (mg/kg)	7.8 ± 2.3	7.4 ± 2.4	7.7 ± 2.2	0.92
FE’ISO (AUC)	23.1 ± 2.2	22.8 ± 1.1	17.8 ± 3.1	0.0004^†^
Anesthesia time (min)	38.6 ± 8.5	46.9 ± 10	42.4 ± 9.9	0.14
Surgery time (min)	22.4 ± 4.2	23.2 ± 4.3	19.8 ± 4.1	0.19
Extubation time (min)	7.8 ± 5.1	7.8 ± 3.2	7.3 ± 3.9	0.23
Recovery time (min)	39 ± 15.2	38 ± 13.7	33 ± 15.1	0.35

^†^ Significantly different from S and R groups (Tukey’s Test, *P* < 0.05)

### Intraoperative isoflurane requirements and cardiovascular response to surgical stimulation

The AUC of FE’ISO was significantly lower (*P* = 0.0004) in the RM (17.8 ± 3.1) compared to S (23.1 ± 2.2) and R groups (22.8 ± 1.2). Hypertension was detected in 4/15 cats in S and R groups during surgical manipulation of the ovaries and uterus (*P* = 0.07). Hypotension occurred in 1/15, 4/15, and 5/15 cats in the S, R, and RM groups, respectively (*P* = 0.18).

Compared with the S and R groups, mean isoflurane requirements in the RM group were decreased by 21.9 and 15.6%, respectively. When specific time points were compared among groups, mean FE’ISO was significantly lower in the RM group compared to the R and S groups at T2, T3 (*P* < 0.0001), and T4 (*P* = 0.0001) (Table 2). Compared to T1, FE’ISO was significantly higher at T2 and T3 in the S group (*P* < 0.0001), and at T2 in the R group (*P* = 0.0016). In the RM group no significant differences were observed in the FE’ISO over time (*P* = 0.62). HR was significantly lower at T2 (*P* = 0.001), T3 (*P* = 0.0006), and T4 (*P* = 0.002) in the RM compared to R and S groups. Similarly, SAP was significantly lower in the RM at T2 (*P* = 0.001) and T3 (*P* = 0.006) compared to the R and S groups. Compared to T1, HR increased at T2, T3 and T4 (*P* < 0.0001) in the R and S groups, and SAP increased at T2 and T3 (*P* < 0.0001) in the R group and at T2, T3 and T4 (*P* < 0.0001) in the S group. No significant differences were recorded in the RM group over time. No significant differences were observed among groups for RR, FE’CO_2_, and esophageal temperature. Intraoperative supplementation with fentanyl was not necessary.

**Table 2:** Mean ± standard deviation of heart rate (HR), systolic arterial blood pressure (SAP) and end-tidal isoflurane concentration (FE’ISO) recorded during anesthesia of cats undergoing ovariohysterectomy treated with local infiltration with physiological saline (0.4 mL/kg; group S, *n* = 15), ropivacaine (1 mg/kg; group R, *n* = 15) and ropivacaine/meloxicam (0.2 mg/kg; group RM, *n* = 15)

Variable	Group	Time point relative to surgical stimulation				
		T1	T2	T3	T4	T5
**FE’ISO (%)**	**S**	1.10 ± 0.15	1.23 ± 0.27*	1.23 ± 023*	1.13 ± 0.14	1.0 ± 0.23
	**R**	1.02 ± 0.10	1.18 ± 0.15*	1.16 ± 0.18	1.10 ± 0.20	0.96 ± 0.15
	**RM**	1.0 ± 0.20	0.99 ± 0.21^†^	0.99 ± 0.11^†^	0.95 ± 0.13^†^	0.90 ± 0.17
**HR (beats/min)**	**S**	128 ± 20	163 ± 29*	165 ± 27*	157 ± 23*	149 ± 23
	**R**	127 ± 14	157 ± 27*	159 ± 31*	156 ± 29*	147 ± 30
	**RM**	113 ± 17	124 ± 30^†^	123 ± 22^†^	124 ± 26^†^	120 ± 24
**SAP (mmHg)**	**S**	94 ± 24	127 ± 27*	123 ± 24*	113 ± 24*	106 ± 26
	**R**	91 ± 13	123 ± 26*	126 ± 20*	110 ± 26	97 ± 22
	**RM**	91 ± 14	95 ± 18^†^	97 ± 20^†^	97 ± 23	92 ± 21

T1, after skin incision; T2, after the clamping of first ovarian pedicle; T3, after the clamping of second ovarian pedicle; T4, after the clamping of the uterine cervix; T5, after the last skin suture was placed

*Significantly different from baseline values (Tukey’s Test, *P* < 0.05). ^†^ Significantly different from S and R groups (Tukey’s Test, *P* < 0.05)

### Postoperative analgesia and sedation

There were no differences among groups in the number of cats receiving rescue analgesia (*P* = 0.17) or in the number of total rescue analgesic interventions during the observational period (*P* = 0.15). A total of 6 cats required rescue analgesia (4 cats in the S group and 1 cat in each of the R and RM groups). In the R and RM groups, each cat received rescue analgesia on one occasion (one dose of morphine each), whereas in the S group two cats received rescue analgesia on two occasions, and two cats received rescue analgesia on one occasion (total of 6 doses of morphine) (Table 3).

**Table 3:** Number of rescue doses administered over time following ovariohysterectomy in cats treated with local infiltration with physiological saline (0.4 mL/kg; group S, *n* = 15), ropivacaine (1 mg/kg; group R, *n* = 15) and ropivacaine/meloxicam (0.2 mg/kg; group RM, *n* = 15). The total number of cats that received postoperative analgesia is also reported

Group	Postoperative period (hours)								Total number of rescue doses	Total number of rescued cats
	0.5	1	2	4	6	8	12	24		
S	0	2	3	1	0	0	0	0	6	4/15
R	0	1	0	0	0	0	0	0	1	1/15
RM	0	0	0	1	0	0	0	0	1	1/15

The AUC recorded for the IVAS in the S, R and RM groups was 142 ± 59, 141 ± 57 and 119 ± 75, respectively. The AUC of MCPS was 20 ± 13 (S group), 21 ± 13 (R group), and 14 ± 9 (RM group). For the MNT, AUC (0.5–24 h) values were 4718 ± 503, 4665 ± 1440, 4613 ± 1538. The AUC of the IVAS, MCPS and MNT did not differ among groups (*P* = 0.56, 0.64, and 0.18, respectively).

Group comparisons at each time point showed that lower IVAS pain scores were detected in the first hour after extubation in the RM compared to R and S groups (*P* = 0.021–0.018) (Table 4). The IVAS scores were significantly increased compared to corresponding baseline values from 1 to 24 h in the S group, from 0.5 to 12 h in R group and from 2 to 12 h in the RM group (*P* < 0.001). For the MCPS scores and MNT measurements, no significant differences were observed for either treatment or time (*P* > 0.05).

**Table 4:** Pain and sedation scores [median (range)], and mechanical nociceptive thresholds [mean (SD)] measured prior to ovariohysterectomy (BL) and at 0.5, 1, 2, 4, 6, 8, 18 and 24 h of the postoperative period in cats treated with local infiltration of physiological saline (S), 1 mg/kg of ropivacaine (R) and ropivacaine (1 mg/kg) / meloxicam (0.2 mg/kg) (RM). All groups started with an *n* = 15. Animals that received rescue analgesia were removed from comparisons among treatment groups and from baseline. The actual number of animals analyzed at each time point is presented

Variable	Group	Postoperative time (hours)									
		BL	0.5	1	2	4	6	8	12	18	24
Number of animals analyzed	S	15	15	15	13	11	11	11	11	11	11
	R	15	15	15	14	14	14	14	14	14	14
	RM	15	15	15	15	15	15	14	14	14	14
IVAS (0-100 mm)	S	0 (0–0)	5 (0–10)	5 (0–35)*	5 (5–35)*	10 (0–25)*	5 (0–20)*	5 (0–20)*	5 (0–15)*	5 (0–10)*	5 (0–10)*
	R	0 (0–0)	5 (0–15)*	10 (0–20)*	5 (0–20)*	5 (0–15)*	5 (5–15)*	5 (0–20)*	5 (0–10)*	5 (0–10)	5 (0–10)
	RM	0 (0–0)	5 (0–10)	5 (0–10)^†^	5 (0–20)*	5 (0–35)*	5 (0–20)*	5 (0–15)*	5 (0–20)*	5 (0–10)	5 (0–15)
MCPS (0–24 point)	S	0 (0–5)	0 (0–2)	0 (0–15)	1 (0–7)	1 (0–4)	1 (0–5)	1 (0–5)	1 (0–2)	1 (0–2)	0 (0–2)
	R	0 (0–3)	0 (0–2)	1 (0–7)	0.5 (0–2)	1 (0–4)	1 (0–5)	1 (0–4)	1 (0–2)	1 (0–2)	0.5 (0–2)
	RM	0 (0–5)	0 (0–4)	0 (0–1)	0 (0–5)	0 (0–6)	0.5 (0–5)	1 (0–2)	1 (0–2)	0 (0–2)	0.5 (0–2)
MNT (grams)	S	211 ± 56	237 ± 92	196 ± 64	196 ± 63	188 ± 84	185 ± 51	185 ± 74	184 ± 56	183 ± 27	190 ± 28
	R	288 ± 60	173 ± 87	183 ± 59	167 ± 70	142 ± 67	163 ± 61	166 ± 77	185 ± 78	180 ± 64	181 ± 64
	RM	223 ± 94	280 ± 95	215 ± 93	205 ± 98	163 ± 64	156 ± 39	160 ± 56	210 ± 87	195 ± 83	185 ± 44
Sedation (0–4 point)	S	0 (0–0)	1 (0–3)*	0 (0–1)	0 (0–0)	0 (0–0)	0 (0–0)	0 (0–0)	0 (0–0)	0 (0–0)	0 (0–0)
	R	0 (0–0)	1 (0–3)*	0 (0–1)	0 (0–0)	0 (0–0)	0 (0–0)	0 (0–0)	0 (0–0)	0 (0–0)	0 (0–0)
	RM	0 (0–0)	1 (1–3)*	0 (0–1)	0 (0–0)	0 (0–0)	0 (0–0)	0 (0–0)	0 (0–0)	0 (0–0)	0 (0–0)

*IVAS* Interactive Visual Analogue Scale, *MCPS* UNESP-Botucatu Multidimensional Composite Pain Scale, *MNT* Mechanical Nociceptive Thresholds

*Significantly different from baseline values (*P* < 0.05). ^†^Significantly different from S and R groups (*P* = 0.021–0.018)

Sedation scores did not differ between groups during the 24-h period. When compared with baseline values, increased scores were recorded at 0.5 h after extubation (*P* < 0.001) (Table 4).

### Adverse events

The incidence of hematoma did not differ among groups. Hematoma was observed in the ovarian pedicles after the injection in 3/15, 2/15, and 2/15 cats in the S, R, and RM groups, respectively (*P* = 0.84). No signs of local anesthetic toxicity were observed during the study period.

## Discussion

This study showed that, when compared to infiltration of ropivacaine alone or physiological saline, infiltration of the surgical site with ropivacaine and meloxicam significantly reduced intraoperative isoflurane requirements and the IVAS pain scores during the first hour following ovariohysterectomy in cats. These findings suggest that, although intraoperative nociception appears to be reduced by the drug combination, it may result in a short acting postoperative analgesic effect, partially supporting the hypothesis of this study.

Previous clinical reports have shown an anesthetic-sparing effect of adding different loco-regional anesthetic techniques to general anesthesia in dogs [6] and cats [5, 7]. However, in our study the isoflurane requirements were comparable between cats receiving incisional infiltration with saline solution or ropivacaine; whereas a significant decrease in FE’ISO was detected only in cats receiving ropivacaine and meloxicam. Overall isoflurane requirements were reduced by 22% in cats receiving local infiltration with ropivacaine and meloxicam compared to the saline infiltration. The lower isoflurane concentration used to maintain anesthesia in the RM group suggest an antinociceptive effect of the drug combination. While ropivacaine exerts its antinociceptive effects by blocking the afferent sensory signal transmission [12], meloxicam can reduce the release of peripheral inflammatory mediators, preventing sensitization of the afferent fibers [18]. Ovariohysterectomy involves the surgical manipulation of ovaries that are highly innervated by sensory and autonomic fibers and extremely responsive to noxious stimulation [19]. Traction of the ovarian ligament and clamping of its pedicle triggers an autonomic response, signaled by the increase in physiologic parameters, such as HR and arterial pressure, which have often been used as indicators of nociception in anesthetized patients [7, 19]. Whereas intraoperative hypertension was detected in both S and R groups (27% of the cats), no cats in the RM group presented hypertension, suggesting that the drug combination provides better control of the blood pressure response caused by surgical manipulation of the ovaries and uterus. It is important to emphasize that meloxicam was also administered preoperatively in the S and R groups by subcutaneous route. Thus, it appears that the local infiltration of meloxicam can offer clinical advantages compared to its systemic administration. With local infiltration, higher drug concentration is reached at the point of the origin of the inflammatory process, which could improve the analgesic effect [13, 18]. In humans, the pharmacokinetic parameters of meloxicam demonstrated that its maximum plasma concentration was achieved at 108 min after the local administration in surgical patients with inguinal hernia repair, suggesting that this NSAID was slowly released in the systemic circulation [18]. In view of these results, it is possible that with local infiltration a high concentration of meloxicam was achieved at the surgical site throughout the intraoperative period and during the first hour of the postoperative period, when lower IVAS pain scores were detected in the RM group.

Whether or not the infiltration of a local anesthetic at the surgical wound contributed to control postoperative pain is controversial. Previous clinical reports did not find relevant analgesic effects following the infiltration of bupivacaine or lidocaine at the incision site for the control of pain after ovariohysterectomy in dogs [3, 19] and cats [20]. However, the present study differs from these previous reports because, besides the midline incision site, the local anesthetic was also infiltrated in other anatomical areas involved in the surgical trauma caused by ovariohysterectomy, including the ovarian pedicles and the uterine cervix. Fudge et al. [8] used a similar technique of tissue infiltration with bupivacaine and found low postoperative pain scores in cats undergoing ovariohysterectomy. Similarly, intraperitoneal instillation of bupivacaine or bupivacaine-dexmedetomidine on each ovarian pedicle and caudal uterus provided effective postoperative analgesia after ovariohysterectomy in cats [4, 21]. Nevertheless, in the present study, except for the first hour post-extubation, when lower IVAS scores were identified in the RM group, no significant differences among groups were found in postoperative pain assessments. These results are consistent with a previous report in rats, where local infiltration of ketorolac in combination with dexamethasone reduced postoperative pain scores only until 2 h after laparotomy, which could be attributed to the systemic absorption of drugs provided by the large vascularization of the abdominal cavity [22]. In human surgical patients undergoing hernia repair, no significant analgesic benefits were found with local infiltration of NSAIDs compared to systemic injection of meloxicam [18] or ketorolac [23]. Moreover, some aspects of our experimental protocol may have contributed to the apparent lack of a prolonged postoperative analgesic effect of ropivacaine in combination with meloxicam. This study was designed to investigate the use of local anesthesia, as part of a multimodal analgesic protocol, in cats under general anesthesia. For ethical concerns, and to use a protocol that would approach the clinical practice, all cats received an opioid (pethidine) prior to surgery. Moreover, meloxicam was administered preoperatively in the R and S groups. Despite the short duration of action of pethidine, approximately 1–2 h [24], Lascelles et al. [25] reported that its preoperative administration prevented allodynia and decreased MNT responses in dogs after ovariohysterectomy. In addition, pharmacokinetic studies have reported that meloxicam has an elimination half-life of approximately 24 h in cats following subcutaneous administration [26] and it has been shown to be an effective analgesic for pain relief after feline ovariohysterectomy [16, 27]. Thus, the provision of preoperative analgesics may have influenced pain assessments and reduced the differences among groups. Furthermore, all the surgeries were performed by an experienced surgeon, resulting in minimal tissue trauma, which may have contributed to the low overall pain intensity for most cats (median IVAS < 15 mm and MCPS < 2), making it difficult to establish significant differences among groups. In addition, animals that received morphine during the postoperative period were removed from comparisons of pain scores (IVAS, MCPS and MNT). Notwithstanding removal of rescued animals avoided the bias caused by morphine on postoperative pain scores, it may have reduced the power of the study to detect differences in pain scores among groups.

When considering postoperative analgesic supplementation, few cats (6 out of 45 cats) required rescue analgesia. In the S group, rescue analgesia was given in 4 cats (27%), which is in agreement with previous studies that reported a prevalence of rescue analgesia between 13 and 50% after ovariohysterectomy in cats treated preoperatively with an opioid alone or combined with meloxicam [4, 24]. Although the frequency of recue analgesia did not differ among treatments, only one cat in each R and RM groups required supplemental analgesics, indicating that both protocols of local analgesia provided was some analgesic benefit when compared to saline controls. In the S group, all rescued cats received the first dose of morphine until 2 hours after extubation, which is consistent with previous data reported by Quarterone et al. [17]. In cats with experimentally induced inflammation, the maximum plasma concentration of meloxicam was achieved at 2.2 ± 0.7 h, with the highest decreases in pain scores recorded at 5.2 ± 1 h after subcutaneous administration [26]. These data may explain the high incidence of rescue analgesia in the first 2 h in the S group. In the same period of evaluation, rescue analgesia was given to one cat of the R group (1 h), while the time for the first rescue analgesia was longer (4 h) in the RM group, suggesting that the analgesic effect may have been prolonged by the combination of meloxicam and ropivacaine. However, due to the small number of cats receiving rescue analgesia, this effect cannot be confirmed without a larger sample size.

The most common complications related to the local infiltration of drugs at the incision site include minor wound hematoma, edema, drainage, fluid accumulation, and infection [28]. In the current study, hematoma of the ovarian pedicles was the only wound-related complication, and occurred in few cats (15%), suggesting that the injection technique used was safe. Intraoperatively, hypotension was detected in 22% of the cats. Although the incidence of hypotension did not differ among treatments, it was more frequently observed in cats treated with local analgesia (33, 27 and 7% in the RM, R and S groups). However, higher incidence of intraoperative hypotension (59 to 82%) has been reported using different systemic opioids in isoflurane-anesthetized cats [29, 30]. Thus, it appears that the incisional blockade with ropivacaine alone or combined with meloxicam did not increase the risk of hypotension. Additionally, in all cats the hypotension was transient and was corrected during surgery using only a crystalloid bolus.

This study has some limitations. One potential reason for failure to demonstrate significant differences among groups in the frequency of rescue analgesia and in the MCPS pain scores could be attributed to the small sample size. The sample size was estimated considering a frequency of rescue analgesia of 70% in the S group and 20% in the treatment groups (R and RM). However, the differences in the frequencies of rescue analgesia were smaller than this, limiting the statistical power of our study. Moreover, as the dose and volume play an important role in the action of local anesthetics it is possible that these factors may have interfered in our results. To date, there are no studies concerning the local infiltration of ropivacaine for postoperative pain relief in cats and the pharmacokinetics of this local anesthetic has not been reported in cats. Thus, the dose of ropivacaine administered was based on the veterinary literature in order to not exceed the maximum recommended dose for cats, especially as there is limited information regarding pharmacokinetics and toxic dose of ropivacaine in cats. It is possible that the volume/concentration of ropivacaine administered in this study was insufficient for proper perioperative pain control following ovariohysterectomy in cats.

## Conclusions

As part of multimodal pain therapy, the local infiltration of the surgical site with ropivacaine and meloxicam showed evidence of a superior intraoperative antinociceptive effect than ropivacaine alone, as it significantly decreased the isoflurane requirements for maintaining anesthesia and attenuated the cardiovascular response to surgical stimulation. The combined infiltrative block of ropivacaine and meloxicam also appeared to result in better analgesia during the early postoperative period, but these results were limited by the relatively small sample size. Further studies are needed to determine the efficacy and safety of local infiltration of different doses and concentrations of ropivacaine, and its combination with meloxicam in cats.

## Methods

### Animals

After obtaining informed consent, 45 crossbreed client-owned cats admitted for elective ovariohysterectomy were enrolled. The study was performed following the guidelines of CONCEA (Brazilian Council of Control in Animal Experimentation), and the experimental procedure was approved by the Institutional Animal Care Committee (protocol 3843/2017 -CEUA). Only cats with normal complete blood count and serum chemistry, aged ≥6 months, and with an American Society of Anesthesiologists physical status I (ASA I) were included in the study. The exclusion criteria were: pregnancy, lactation, extreme aggression, body weight < 2 kg, and systemic diseases. The cats arrived at the hospital at least 48 h prior to surgery to allow the observer to become familiar with each animal. Preoperatively, all cats were evaluated by abdominal ultrasonography for confirmation of the absence of pregnancy. Before each experiment, the cats were fasted overnight with free access to water.

### Anesthesia and surgery

All anesthetic procedures were performed by the same anesthetist who was unaware of group allocation. The cats were sedated intramuscularly (IM) with acepromazine^1^ (0.05 mg/kg) combined with meperidine^2^ (6 mg/kg). Fifteen minutes later, an intravenous (IV) 24-gauge catheter was aseptically placed in the cephalic vein. Anesthesia was induced with (IV) propofol^3^ in a sufficient dose to permit the endotracheal intubation. The cats were attached to a non-rebreathing system^4^ and isoflurane^5^ in 100% oxygen (300 mL/kg/min) was administered for maintenance of anesthesia. Animals were allowed to breathe spontaneously throughout the procedure. Body temperature was maintained between 37 °C–38 °C using a heating pad.^6^ Lactated Ringer’s solution^7^ was administered IV at 5 mL/kg^/^h until extubation.

Electrocardiography (lead II), heart rate (HR), oxyhemoglobin saturation (SpO_2_%), and esophageal temperature were continuously measured using a multi-parameter monitor^8^ (DX 2020, Dixtal Biomédica Ind. Com. Ltda., Brazil); respiratory rate (RR), end-tidal carbon dioxide concentration (FE’CO_2_), and end-tidal isoflurane concentration (FE’ISO) were measured by a gas analyzer^9^ (Gas analyzer module VAMOS plus, Dräger do Brazil, Brazil). Before each experiment, accuracy of the gas analyzer was verified with a standard gas mixture (CO_2_: 5 vol%, N_2_O: 70 vol%, O_2_: 24 vol%, and isoflurane: 1 vol%) (White Martins Gases, Brazil). Systolic arterial blood pressure (SAP) was monitored indirectly by a Doppler ultrasound device^10^ (Doppler 841-A; Parks Medical Electronics, USA), using an appropriately sized cuff, between 30 and 40% of the circumference of the thoracic limb, with the probe placed over the digital artery.

The FE’ISO, HR and SAP were recorded at specific time points throughout anesthesia, as follows: T1 = after skin incision, T2 and T3 = after the clamping of first and second ovarian pedicles, respectively, T4 = after the clamping of the uterine cervix, and T5 = after the last skin suture was placed. Ovariohysterectomy was performed using a standard technique through median laparotomy access. All surgical procedures were performed by the same surgeon using a 3-cm ventral midline approach and 3-clamp technique.

Anesthesia time (time elapsed from the administration of propofol to discontinuation of isoflurane), surgery time (time elapsed from the first incision until placement of the last suture), time to extubation (time elapsed from termination of isoflurane until orotracheal tube removal), and recovery time (time elapsed from discontinuation of isoflurane until voluntary movement into a sternal position) were recorded for each cat. Extubation was performed when the cat recovered palpebral reflexes.

### Study design and treatment administration

In a prospective, randomized, blinded, positive-controlled clinical study, cats were randomly assigned using an online software program^11^ to receive one of the three treatments (*n* = 15): S group, local infiltration of physiological saline (0.4 mL/kg); R group, local infiltration of ropivacaine^12^ (1 mg/kg); RM group, local infiltration of ropivacaine (1 mg/kg) combined with meloxicam^13^ (0.2 mg/kg). Meloxicam (0.2 mg/kg) was administered subcutaneously to cats of the S and R groups after orotracheal intubation.

**Fig. 1:**
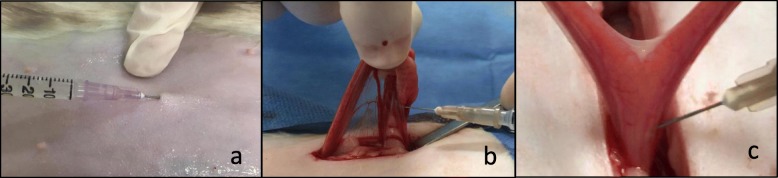
Local infiltration into the ovarian pedicle (**a**), uterine cervix (**b**) and midline incision (**c**). Cats received 0.9% physiologic solution (group S, *n* = 15); 0.25% ropivacaine (1 mg/kg; group R, *n* = 15); or ropivacaine and meloxicam (0.2 mg/ kg; group RM, *n* = 15). The solutions were diluted with saline to a total volume of 0.4 mL/kg and were administered in equal parts into the subcutaneous tissue of the incision site, right and left ovarian pedicles, and caudal uterine body. The same volume of saline solution was administered into the same surgical sites

An identical volume (0.4 mL/kg) was used for local infiltration in all groups. To adjust the same volume in all groups, 1% ropivacaine^l^ was diluted to a 0.25% solution with physiological saline in the R group. In the RM group, 0.2 mg/kg of 0.2% meloxicam^m^ was used to complete the volume of physiological saline necessary to dilute 1% ropivacaine to a 0.25% solution. The total volume administered to each cat was equally divided into four 1 mL syringes attached to 12.7 mm long, 28 Gauge needles for infiltration into four specific anatomical areas (subcutaneous tissue of the skin incision site, right and left ovarian pedicles, and caudal uterine body). To avoid accidental puncture of abdominal structures, the ovarian pedicles and caudal uterine body were fully exposed by the surgeon, who aseptically all infiltrations (Fig. 1).

Before the beginning of surgery, the cats received the incisional subcutaneous infiltration with one of the three treatments. After the abdomen was surgically opened and the uterus and ovaries were exposed, solutions were infiltrated in the ovarian pedicles (left and right) and uterine body caudal to the bifurcation. Ten minutes later, the excisions of the pedicles and uterus were initiated.

### Evaluation of intraoperative isoflurane requirements and cardiovascular responses to surgical stimulation

At the beginning of anesthesia, the vaporizer was set at 1% isoflurane. Intraoperatively, vaporizer settings were adjusted to maintain surgical depth of anesthesia (loss of palpebral reflexes and loss of jaw tone) and to prevent autonomic responses to surgical stimulation.

Heart rate and SAP were continuously monitored throughout anesthesia. If SAP or HR increased by more than 20% from the previously recorded values during surgical stimulation, FE’ISO was increased by 0.2%. If an FE’ISO above 1.6% was required, additional analgesia was provided with fentanyl (1 μg/kg, IV). Bradycardia, hypertension, and hypotension lasting ≥5 min (defined as a HR < 90 beats minute^− 1^, SAP < 90 mmHg, and SAP > 160 mmHg, respectively) were recorded. Bradycardia was treated with IV atropine (0.022 mg/kg), as required. Hypotension was treated with IV crystalloid bolus (10 mL/kg lactated Ringer’s, for 10 min, repeated if necessary). In case of hypotension non-responsive to fluids, IV dopamine (5–10 μg/kg/min), was administered.

### Assessment of post-operative pain and sedation

The same single observer, unaware of the treatment groups, was responsible for pain and sedation assessments, which were performed 24 h prior to surgery (baseline), and 0.5, 1, 2, 4, 6, 8, 12, 18, and 24 h after extubation. The observer was a veterinary graduate student, with previous training in the assessment of pain in cats using pain scales. Pain was assessed by two different pain scoring systems, including the Interactive Visual Analogue Scale (IVAS, from 0 mm = no pain to 100 mm = maximum pain) and UNESP-Botucatu Multidimensional Composite Pain Scale (MCPS, from 0 = no pain to 24 = maximum pain). The MCPS pain scoring involved only two domains (pain expression, scale range = 0–12 points; psychomotor change, scale range = 0–12 points) [31]. For scoring, each cat was initially evaluated for 1 min in its cage. Following this, the cat was stimulated to move around, for observation of reactions and behaviour. Finally, the abdomen and the area surrounding the abdominal incision was palpated using 2–3 digits, and the reaction of the cat was assessed and recorded.

The pain scores were also assessed with mechanical nociceptive thresholds (MNT) using an electronic von Frey device.^14^ For the MNT testing, the peak force exerted by the tip of the electronic von Frey device was recorded in grams (maximum 700 g). The tip was applied with the cats in lateral recumbency, approximately 1 cm from the surgical wound, at three points: cranial, caudal, and lateral. The final MNT was the median of the three recorded values. The device was removed immediately if the cat exhibited signs of pain, such as withdrawal movement, contraction of the abdominal wall, attempts to bite/scratch, and vocalization. The MNT was assessed after the IVAS and MCPS measurements at the same time points.

Morphine^15^ was administered (0.1 mg/kg IM) as rescue analgesia if the MCPS scores were ≥ 6, as reported by previous studies [4, 21]. The number of cats requiring rescue analgesia and the number of morphine doses were recorded.

A numerical rating score was used for the assessment of the degree of sedation, were: 0 = completely awake, able to stand and walk; 1 = stands, but staggers when attempting to walk; 2 = with encouragement is unable to stand but laying in sternal recumbency with head elevated; 3 = able to lift head with encouragement, but resting head down, sternal recumbency; 4 = responsive to light stroking, lateral recumbency; 5 = unresponsive to light stroking, lateral recumbency [32].

### Adverse events

The occurrence of complications related to the injection at the surgical site, such as hemorrhage during surgery, accidental intravascular injection, and hematoma formation were recorded. Other adverse events during the study period such as seizures, nausea, vomiting, were also recorded. After hospital discharge, cats were evaluated at home until surgical stitch removal, and the owners were instructed to report the occurrence of behavioral changes and signs of adverse events, such as vomiting, diarrhea, anorexia, lethargy, melena, polydipsia, polyuria, adipsia.

### Outcome measures

The primary outcome measures were the isoflurane requirements for maintaining anesthesia, postoperative pain scores/MNT and the requirement for the postoperative rescue analgesia. Secondary outcome measures included the sedation scores and adverse events.

### Statistical analysis

A sample size of at least 15 cats per group was estimated to achieve 80% statistical power to detect a prevalence of a postoperative treatment failure of 70% in the S group and 20% in the treated groups (R and RM). Sample calculation was based on pilot data.

A Shapiro-Wilk test was performed to assess the normality of data distribution. Data are expressed as mean ± standard deviation (parametric variables) or median (range) (non-parametric variables) where appropriate.

Bodyweight, age, dose of propofol, time to extubation, and surgical, anesthetic, and recovery times were compared among groups using one-way ANOVA followed by a Tukey’s test.

The area under the curve (AUC) of FE’ISO was calculated from T1 until T5 using the trapezoidal method and compared among groups using ANOVA and a Tukey post-test. Values of FE’ISO, HR and SAP recorded intraoperatively were compared among groups by a two way-ANOVA followed by Tukey post-hoc test. Differences between baseline and other time points within each group were assessed by repeated measures ANOVA and a Tukey post-test.

The incidence of adverse events in the three groups and the number of cats that required rescue analgesia post-operatively was compared among groups using a chi-square/Fisher’s exact test. A Kruskal-Wallis test compared the number of morphine doses administered post-operatively among groups. After receiving the first dose of rescue analgesia cats were removed from the statistical analysis of IVAS, MCPS, MNT and sedation scores. Corresponding AUCs of IVAS, MCPS and MNT values were calculated from baseline until 24 h using the trapezoidal method. A Kruskal-Wallis test compared pain (IVAS and MCPS) and sedation scores among groups (data with asymmetric distribution). A Friedman test compared differences in pain and sedation scores over time within each group**.** The MNT measurements were compared among groups using two-way ANOVA and a Tukey post-test. (data with symmetric distribution).

The analyses were performed using GraphPad Prism7.0^16^ (GraphPad Software Inc., CA, USA) and R software version 3.5 (https://www.r-project.org/). Differences were considered significant when *P* < 0.05.

## Data Availability

The datasets used and/or analyzed during the current study available from the corresponding author on reasonable request.

## Abbreviations

IVAS: Interactive visual analog scale
MCPS: UNESP-Botucatu multidimensional composite pain scale
MNT: Mechanical nociceptive thresholds

## Footnotes

1 Acepran 0.2%, Vetnil, São Paulo, Brazil.

2 Dolosal, Cristália, São Paulo, Brazil.

3 Propovan 1%, Cristália, São Paulo, Brazil.

4 SAT 500, Takaoka, São Paulo, Brazil

5 Isoforine, Cristália, São Paulo, Brazil.

6 Colchão Térmico, Brasmed, São Paulo, Brazil.

7 Ringer com Lactato, JP Pharmaceutical Ind., Ribeirão Preto, Brazil.

8 DX 2010; Dixtal, São Paulo, Brazil.

9 Gas analyzer module VAMOS plus, Dräger do Brazil, Barueri, Brazil

10 Doppler 841-A; Parks Medical Electronics, OR, USA.

11 Research Randomizer, Computer software, http://www.randomizer.org/, Pennsylvania, USA

12 Ropi 10 mg/mL, Cristália, São Paulo, Brazil.

13 Maxicam 0.2%, Ourofino Saúde Animal, São Paulo, Brazil.

14 Electronic von Frey anesthesiometer, IITC Life Science, CA, USA

15 Dimorf injetável 10 mg, Cristália, São Paulo, Brazil.

16 GraphPad Software Inc., CA, US
